# Specialization of Physician Associates and Nurse Practitioners as Reflected in Workforce Projections

**DOI:** 10.7759/cureus.73216

**Published:** 2024-11-07

**Authors:** Roderick S Hooker, Kim Curry, Christine Tracy

**Affiliations:** 1 Health Services Research, Retired, Portland, USA; 2 Nursing, University of Florida, Tampa, USA; 3 Biostatistics, American Association of Nurse Anesthesiology, Rosemont, USA

**Keywords:** graduate education, health workforce, nurse practitioner, physician assistant, physician associate, specialization, workforce

## Abstract

Introduction

The utilization of physician associates (PAs) and nurse practitioners (NPs) within the United States healthcare system has increased substantially since their inception in the 1960s. While many PAs and NPs are educated and work within specialty areas in their clinical practice, the federal government does not identify PAs and NPs by specialty in its workforce projections.

Methods

We obtained data from publicly available sources as well as professional organizations. Employment status, specialty, and location information for NPs and PAs were drawn from association census surveys.

Results

Outpatient or ambulatory care employment sites employ 46.6% of NPs and 55.3% of PAs, while almost a quarter of each group (22.1% and 23.9%, respectively) work in general and surgical hospitals. About 9% of providers who are PAs and NPs are employed in Federally Qualified Health Centers, many of which are in health professional shortage areas (HPSAs). The number of PAs and NPs working in HPSAs has significantly increased from 72,000 PAs and NPs in 2011 to 110,000 in 2023. There has been no growth of physicians in HPSAs during this same time. The Health Resources and Services Administration (HRSA) does not identify NPs and PAs by their specialty practice area so the extent to which they provide care in specialty areas is unknown based on HRSA data.

Conclusion

It is essential to provide detailed information inclusive of PAs and NPs as well as physicians in planning for healthcare workforce needs. The accurate projection of potential healthcare provider shortages requires knowledge of each type of provider along with their role and specialization to determine gaps in healthcare service delivery.

## Introduction

The first educational programs for nurse practitioners (NP) and physician associates/assistants (PA) began in the mid-1960s [1, 2]. For over 50 years, the roles of these two types of health professionals have grown in numbers, scope of practice, and degree of clinical specialization. According to the Bureau of Labor Statistics (BLS), PA and NP providers represent a third of all clinically active medical providers in the United States [3]. Where they are employed and how they are utilized is known primarily through medical and surgical specialty reports and commercial data sources. These reports are generated mainly by labor economists and health services researchers acting independently of the federal government and focusing on forecasting areas of specialization [4]. This includes specialty areas such as orthopedics, psychiatry, critical care, pediatrics, and many others [5, 6].

Despite significant employment growth, personal and professional characteristics of PAs and NPs are often absent from key publicly available data sources. For example, the Health Resources and Services Administration (HRSA) periodically examines where physician workforce shortages are predicted. These predictions address communities with the most need for physician specialties while listing PAs and NPs as generic providers rather than including their specialist roles. Physician specialties such as psychiatry, cardiology, and gynecology in health professional shortage areas (HPSAs) are counted in the HRSA census. In contrast, the specialty information for PAs and NPs is omitted.

Within HRSA, the National Center for Health Workforce Analysis Area Health Resource File identifies 16 physician specialties but omits any PA or NP specialty [7]. The Bureau of Labor Statistics (BLS) has the same clinician filter in place, delineating physicians but omitting the specialties of NPs and PAs. According to HRSA, in 2023, approximately 41,000 PAs and 69,000 NPs worked in HPSAs [7]. This represents a significant increase from 2011, when only 29,000 PAs and 43,000 NPs worked in HPSAs. There has been no growth of physicians in HPSAs during this same time.

The Association of American Medical Colleges (AAMC) also collects data on physicians to project workforce needs. The AAMC recognizes the increasing utilization of PAs and NPs impacting physician demand and factors in their forecasting models [5]. Unlike the BLS, the AAMC addresses the specialization demand in some areas with PAs and each type of advanced practice registered nurse (APRNs): nurse practitioners, certified nurse midwives, nurse anesthetists, and clinical nurse specialists. In projecting physician workforce demands through 2036, the overall impact is expected to be a shortage of 38,900 to 78,000 physicians, depending on the modeling scenario [5]. In addition, the AAMC projections model three scenarios related to the type of delivery system: maintenance of the status quo, a scenario making heavy use of retail clinics staffed by NPs and PAs for routine care, and a population health scenario with added focus on prevention and chronic disease management. The AAMC concludes that physician shortages will be smaller than previously expected. This conclusion is based on the assumption that graduate medical education funding will grow and not a reliance on the growing presence of NPs and PAs.

In addition to problems with variation in how data are collected and shared, funding has been mentioned as one factor in omitting more specific and inclusive data for NP and PA providers. Under the Affordable Care Act, a national healthcare workforce commission was created to study and recommend improvements to the entire workforce, including NPs, PAs, and others. However, Congress did not authorize funding for this to occur [8]. Such omissions handicap predictive modeling specific to national healthcare provider needs [5]. This is particularly important in forecasting future healthcare workforce shortages, as these forecasts are only accurate if all potential providers are part of the equation [9].

The objective of this study was to examine the specialization of Physician Associates (PAs) and Nurse Practitioners (NPs) and the implications for workforce projections in healthcare. Our study analyzes employment, specialization, and impact of federal workforce projections for PAs and NPs. Understanding the specialization of PAs and NPs is essential for accurate workforce planning to address healthcare provider shortages.

The following terms are defined for the purposes of this report:

Healthcare provider: a person qualified to assume the overall responsibility for the medical care of a person or group of people.

Specialization: The clinical specialty area. Examples include cardiac, orthopedics, pediatrics, psychiatry, and others.

Scope of practice: The extent of patient care activities that the healthcare provider is educated and trained to provide.

Employment setting: The type of care setting in which the provider works. Examples include outpatient clinics, hospitals, and others.

Workforce projections: Projections of the future supply and demand, including growth or shrinkage in the market or among types of healthcare providers.

## Materials and methods

Methods

We obtained data from publicly available sources as well as professional organizations. Table 1 and Table 2 provide details of the data sources. Data of interest included professional role (NP or PA), demographic information, area of specialization, location of employment, and education. Employment status, specialty, and employment location information for NPs and PAs were drawn from association census surveys, including the American Association of Nurse Practitioners (AANP) and National Commission on the Certification of PAs (NCCPA) [10, 11]. Employment, employer type, age, and gender were obtained through the federal Health Resources and Services Administration Bureau of Labor Statistics (BLS) and the US Census Bureau [12].

**Table 1:** Sources of workforce statistics for NPs and PAs

Organization	Source	Data
American Association of Nurse Practitioners	Quadrennial survey	Education, Primary specialty, Employment
American Academy of Physician Associates	Annual registry	Education, Employment
National Commission on Certification of Physician Assistants	Annual registry	Education, Primary specialty, Employer
Bureau of Labor Statistics	Occupational employment, wage and workforce projections	Employment location, Workforce projections, Wages
American Association of Colleges of Nursing	Commission on Collegiate Nursing Education	Education, Educational programs
Accreditation Review Commission on Education for the Physician Assistant	Accreditation reports	Education, Educational programs
Association of American Medical Colleges	Data and Reports	Workforce projections
Physician Assistant Education Association	Resources	Educational programs
United States Census Bureau	2020 Decennial census	Population statistics

**Table 2:** Data sources for NP and PA characteristics BLS: Bureau of Labor Statistics: (www.bls.gov) AANP: American Association of NPs (www.aanp.org) NCCPA: National Commission on the Certification of PAs (www.nccpa.net) AACN: American Association of Colleges of Nursing (www.aacnnursing.org) PAEA: PA Education Association (paeaonline.org)

Characteristic	Source
Age	BLS, NCCPA
Gender	BLS
Location of employment	BLS
Education	AANP, NCCPA
Employer	AANP, NCCPA
Specialty	AANP, NCCPA
Education track	NCCPA, AACN, PAEA
# of graduates	NCCPA, AACN, PAEA

A variety of data sources were available to assess various aspects of the status and employment of PAs and NPs in the United States. However, no comprehensive source exists. Thus, we examined 14 different data sources to provide information pertinent to this analysis. Additionally, we included educational and licensure data to ensure that readers understand the length of educational programs for these providers as well as the requirements to sit for a licensure examination after completion of the graduate academic program. The most recent data from each source were included. Data obtained from AANP and NCCPA reflect statistics collected during 2022. Other data sources reflect 2023 data.

## Results

As of May 2023, 280,140 NPs and 145,740 PAs were employed in clinical roles (Table 3). The majority were female, with 251,565 (89.8%) of NPs and 100,269 (68.8%) of PAs reporting female gender. The median age was 43 and 38, respectively.

**Table 3:** Overview of the NP and PA workforce Source: Bureau of Labor Statistics, 2023

Characteristic	NP	PA
Total employed (Est.)	280,140	145,740
Employed per 100,000 (Est.)	77.5	42.2
Female (%)	251,565 (89.8)	100,269 (68.8)
Male (%)	28,574 (10.2)	45,470 (31.2)
Median age	42.7	38.3

The work setting for NPs and PAs is identified by the industry in which they are employed. Major settings of employment for NPs and PAs are depicted (Table 4). The term “Office of Physicians” is used by the BLS to indicate outpatient or ambulatory care employment. It is the employment site for 46.6% of NPs and 55.3% of PAs. Next, almost a quarter of each profession (22.1% and 23.9%, respectively) worked in general and surgical hospitals. PAs and NPs (9.0-9.3%) were generically identified as employed in Federal Qualified Health Centers. Many of the federally supported community health centers were in HRSA-designated shortage areas (HPSA) or medically underserved areas [13].

**Table 4:** Employment settings of NPs and PAs Source: Bureau of Labor Statistics, 2023

Employment Setting	NP (%)	PA (%)
Offices of Physicians/Private Ambulatory Setting	137,030 (46.6)	80,610 (55.3)
Hospitals, General & Surgical	61,740 (22.1)	34,890 (23.9)
Outpatient Care Centers	25,440 (9.0)	14,060 (9.3)
Offices of Other Health Practitioners	7,410 (2.6)	2,880 (2.0)
Home Health Care Services	8,234 (2.8)	146 (0.1)
Psychiatric & Substance Abuse Hospitals	2,646 (0.9)	292 (0.2)

The most common specialties self-reported by NPs and PAs are featured in Table 5. Family medicine, primary care, and urgent care are the three most common specialties for NPs. Family medicine, emergency medicine, and orthopedics are the three largest specialties for PAs. These data are collected by professional organizations, typically for use by policymakers, economists, and human resource managers [14].

**Table 5:** Most common specialties of NPs and PAs Source: AANP (2023) [15]; NCCPA (2022) [11]. NOTE: Data above reflect both full-time and part-time clinically practicing NPs and PAs. N/A = not available. There is inconsistency and overlap of provider specialties between surveys, making some specialties unavailable for comparison. The specialties listed reflect areas where 2% or more of either NPs or PAs are employed.

Medical or Surgical Specialty	% of all NPs	% of all PAs
Behavioral/Addiction Medicine	2.2	0.5
Adolescent medicine	N/A	0.1
Cardiology	4.3	2.0
Critical Care Medicine (CCU or ICU)	2.4	2.0
Dermatology	0.8	4.2
Emergency medicine	2.5	11.2
Family Medicine/General Practice	17.9	17.1
Geriatrics	3.2	N/A
Hospice and palliative medicine	2.1	0.2
Hospital medicine/Hospitalist	2.8	3.7
Internal medicine - general practice	4.1	4.1
Internal medicine subspecialties	N/A	7.9
Obstetrics and gynecology	3.1	1.2
Oncology/Hematology	3.2	N/A
Orthopedics	1.7	11.2
Pediatrics-general practice	2.5	1.8
Primary Care	9.8	N/A
Psychiatry	6.1	2.2
Surgery-general	2.8	3.1
Surgical subspecialties	N/A	7.6
Urgent Care	6.4	N/A
Urology	N/A	1.0

Education of PAs and NPs

Current education and licensure status is included below to provide updated information for consideration of NPs and PAs as qualified healthcare providers. Table 6 presents the education tracks for NPs and PAs. Both professions require graduate degrees, including a clinically focused master’s (MSN) or Doctor of Nursing Practice (DNP) for NPs and master’s degrees such as the Master of Physician Assistant Studies (MPAS) for PAs. A clinical doctorate, the Doctor of Medical Sciences (DMSc), is a postgraduate option for PAs [16].

**Table 6:** Education, enrollment, and graduation Sources: AACN 2023; PAEA, 2023; NCCPA/ARC PA, 2023 Note: * = approximate

Component	NPs	PAs
Accredited education programs (master's or doctoral)	650	303
Total number of graduates	39,000*	11,000*
Mean number of graduates per program	60	36

The Commission on Collegiate Nursing Education currently certifies 650 master's or DNP-level programs preparing nurse practitioners (J. Butlin, personal communication, September 3, 2024). There are currently 303 active PA programs [17, 18]. Almost all PA and NP programs are located within universities. In the case of NPs, colleges of nursing typically have multiple NP specialty tracks available from which prospective students can select.

NP Specialty Education

According to the APRN Consensus Model [19], NP education is differentiated into six population foci. Graduates of NP programs must then pass a national certification exam to become board-certified in their population focus area. Nurse practitioner program tracks include: Family health (across the lifespan), Pediatrics (either acute or primary care), Neonatology, Adult-Gerontology (either acute care or primary care), Psychiatric/mental health (across the lifespan), and Women’s/gender-related healthcare.

On average, the program length for a registered nurse pursuing a master's level NP degree is 24 to 36 months full-time or longer if part-time and three to four years when pursuing a post-baccalaureate DNP [20]. Programs certified by the Certification Commission for Nursing Education adhere to national curricular content standards from the National Organization of Nurse Practitioner Faculties (NONPF) “Criteria for Evaluation of Nurse Practitioner Programs,” established by NONPF’s National Task Force on Quality Nurse Practitioner Education [21].

PA Specialty Education

All PAs are educated in general medicine, and the programs must adhere to a standardized model to maintain accreditation. Thus, there is only one PA program track, in contrast to the six available NP program tracks. The PA program track is General Medicine/Family Medicine.

Educational programs for physician associates are full-time and follow a singular graduate degree pathway formatted by the Accreditation Review Commission on PA Education (ARC-PA). Physician associate programs are 32-40 months long, with approximately half didactic and half clinical education [22]. Graduates of PA programs must sit for a national certification examination to become board-certified. The number of PA programs is predicted to grow to 335 by 2027 [22].

Licensure and Postgraduate Training

Licensure for physicians, NPs, and PAs occurs at the state level. For NPs and PAs, graduation from an accredited program and national certification are required when applying for a license to practice. All NPs must additionally comply with the state’s Nurse Practice Act. By comparison, PA practice is typically defined within one or more sections of the Medical Practice Act, which also covers physician practice at the state level. Statutes specify supervision or collaborative requirements with a licensed physician in the remaining states where these requirements still exist [23, 24]. Postgraduate (PG) clinical training emerged in the early 1970s for PAs and in the 1990s for NPs [21]. These are optional programs beyond graduation and certification and include specialty training. Formal postgraduate training for both roles is a growing trend, but it has yet to be standardized [25, 26].

## Discussion

An estimated 280,140 NPs, 145,740 PAs, and 816,900 physicians and surgeons were clinically employed in the United States (US) as of 2023 [3]. NPs comprise 21.2% of the clinician workforce, PAs 11.6%, and physicians and surgeons 67.1%. Together, they work across the entire range of medical and surgical specialty areas. The addition of approximately 425,880 clinically active PAs and NPs to the existing 816,900 physicians has significantly impacted the ability to meet the growing demand for healthcare services. As a group, NPs and PAs now account for a third of all available clinically active providers, and play a critical role in primary care. For example, most family practice physicians now work with PAs, NPs, or both [6]. Both PAs and NPs are also increasingly essential care providers for the growing older adult population [27].

The demographics of NPs and PAs are more similar than different. Both groups are predominantly female (89% for NPs and 69% for PAs). Most are trained in primary care. A significant percentage of PAs (48%) and NPs (54%) practice in medical offices, followed by general medical and surgical hospitals (24% and 23%, respectively). Both professions have continually evolved and elevated their educational standards, as have allopathic (MD) and osteopathic (DO) educational programs. An example of this is the push for a clinical doctoral degree (DNP) as the entry-level degree for NP practice [20]. This study contributes to the literature through its use of comprehensive national datasets, which provide a broad view of the employment landscape for PAs and NPs. By including data from both government and professional organizations, this study offers a more complete picture of these professionals' roles in the healthcare system.

Implications for practice

Adaptability to changing needs, especially in primary care and underserved areas, makes PAs and NPs optimal responders to market demands in a continuously evolving healthcare environment. Constraining roles and scope of practice are emerging as contributors to healthcare costs by artificially restricting the supply of medical providers, and this can increase the financial burden of care by confining the supply of workers. Further work is needed to ensure a broad understanding of the usefulness of NPs and PAs in the healthcare marketplace.

There is a growing need to understand the changing specialties and scope of practice of NPs and PAs. These professions make up a growing percentage of the healthcare provider workforce. Stakeholders in NP and PA professional organizations should work with their associations and policymakers to ensure the collection of comprehensive and consistent data that reflects updated information that can be used to identify the true extent of healthcare provider shortages. Failure to account for the rapidly growing number of PA and NP providers, such as in HRSA calculations of health professional shortage areas, may create inaccuracies in projections of the country’s healthcare supply and demand [8].

Limitations

Historically, self-reported survey data have been a source of information hoped for as reliable, but when survey returns are small, the dependability fades. Professional association surveys also suffer from diminishing rates of return, potential differential response rates across groups, and inadequate representation of small groups. Employer data, as collected from the BLS, also has weaknesses as it needs more means of adjustment to account for dual-employed clinicians (a statistic that may be as high as one-sixth of all PAs) [28]. Employment as a PA or an NP at two or more locations could also confound the actual number.

Because much of the data obtained from professional organizations is self-reported, this introduces the potential for bias and inaccuracy in the data. Nonparticipants in surveys may also constitute a significant bias of omission in association data. In addition, the data presented are necessarily incomplete due to the exclusion of specialty data collection for NPs and PAs. Each of these limitations affects the generalizability of the results.

## Conclusions

Both PAs and NPs are employed across the healthcare continuum in primary care, acute care, and a wide range of specialty areas. While PAs and NPs differ in some ways, they represent an increasing percentage of providers. Consumers, policymakers, and clinicians should consider all qualified providers who can meet the nation's growing needs for healthcare. Projections for the growth of the PA and NP professions reflect their increasing integration into the interprofessional teams that now characterize today’s healthcare system. Accuracy of forecasting of the medical workforce, including potential shortage areas, will improve with consistent, centralized information on all healthcare providers.

## References

[1] Fairman J (2002). The roots of collaborative practice: nurse practitioner pioneers' stories. Nurs Hist Rev.

[2] Cawley JF, Cawthon E, Hooker RS (2012). Origins of the physician assistant movement in the United States. JAAPA.

[3] (2024). Occupational Employment and Wage Statistics. Wage Statistics: IEWS Data.

[4] Hooker RS, Christian RL (2023). The changing employment of physicians, nurse practitioners, and physician associates/assistants. J Am Assoc Nurse Pract.

[5] GlobalData Plc (2024). New AAMC report shows continuing projected physician shortage. Association of American Medical Colleges.

[6] Dai M, Ingham RC, Peterson LE (2019). Scope of practice and patient panel size of family physicians who work with nurse practitioners or physician assistants. Fam Med.

[7] (2024). Area health resource files. https://data.hrsa.gov/topics/health-workforce/ahrf.

[8] Buerhaus PI, Retchin SM (2013). The dormant National Health Care Workforce Commission needs congressional funding to fulfill its promise. Health Aff (Millwood).

[9] Zwilling J, Wise B, Pintz C (2023). U.S. primary care provider needs: an analysis of workforce projections and policy implications. Policy Polit Nurs Pract.

[10] Tracy Tracy, C. C. (2024). The State of the Nurse Practitioner Profession, 2020: Results from the National Nurse Practitioner Sample Survey. American Association of Nurse Practitioners.

[11] National Certification Commission for Physician Associates (2024). Statistical Profile of Board Certified PAs: Annual Report. 2022 Statistical Profile of Board Certified PAs: Annual Report.

[12] (2024). United States Census Bureau: Profiles. https://data.census.gov/profile?q=United%20States&g=010XX00US.

[13] Ku L, Frogner BK, Steinmetz E, Pittman P (2015). Community health centers employ diverse staffing patterns, which can provide productivity lessons for medical practices. Health Aff (Millwood).

[14] Morgan P, Strand De Oliveira J, Short NM (2011). Physician assistants and nurse practitioners: a missing component in state workforce assessments. J Interprof Care.

[15] (2023). 2022 Nurse Practitioner Practice Report. https://storage.aanp.org/www/documents/no-index/research/TOC_practice.pdf.

[16] Kayingo G, Kibe L, Venzon A, Gordes KL, Cawley JF (2022). Assessing demand for doctoral-prepared PA faculty: a five-year longitudinal study. BMC Med Educ.

[17] (2024). Accreditation Standards for PA Education: Fifth Edition. https://www.arc-pa.org/wp-content/uploads/2024/07/Standards-5th-Ed-July-2024.pdf.

[18] (2024). American Association of Colleges of Nursing (2023): 2022-2023 Enrollment and Graduations In Baccalaureate and Graduate Programs in Nursing Washington, DC. https://www.aacnnursing.org/news-data/research-data-center/annual-surveys.

[19] (2024). National Council of State Boards of Nursing. APRN Consensus Model. https://www.ncsbn.org/nursing-regulation/practice/aprn.page.

[20] McCauley LA, Broome ME, Frazier L (2020). Doctor of nursing practice (DNP) degree in the United States: reflecting, readjusting, and getting back on track. Nurs Outlook.

[21] (2024). American Association of Colleges of Nursing. Standards For Accreditation of Baccalaureate and Graduate Nursing Programs. Nursing Education.

[22] (2024). Accreditation Review Commission on Education for the Physician Assistant. Entry level accreditation. https://www.arc-pa.org/entry-level-accreditation.

[23] McMichael BJ, Markowitz S (2023). Toward a uniform classification of nurse practitioner scope of practice laws. Med Care Res Rev.

[24] Valentin VL, Najmabadi S, Jones J, Everett CM (2020). State scope of practice laws: an analysis of physician assistant programs and graduates. J Physician Assist Educ.

[25] Grabenkort R, Hilton G (2022). PA clinical postgraduate training: a half-century of development. JAAPA.

[26] Kidd VD, Vanderlinden S, Hooker RS (2021). A National Survey of postgraduate physician assistant fellowship and residency programs. BMC Med Educ.

[27] Auerbach DI, Levy DE, Maramaldi P, Dittus RS, Spetz J, Buerhaus PI, Donelan K (2021). Optimal staffing models to care for frail older adults in primary care and geriatrics practices in the US. Health Aff (Millwood).

[28] Jeffery C, Morton-Rias D, Rittle M, Cannon J, Hooker RS (2017). Physician assistant dual employment. JAAPA.

